# Mutation-Derived Long Noncoding RNA Signature Predicts Survival in Lung Adenocarcinoma

**DOI:** 10.3389/fonc.2022.780631

**Published:** 2022-03-15

**Authors:** Longjun Yang, Guangran Guo, Xiangyang Yu, Yingsheng Wen, Yongbin Lin, Rusi Zhang, Dechang Zhao, Zirui Huang, Gongming Wang, Yan Yan, Xuewen Zhang, Dongtai Chen, Wei Xing, Weidong Wang, Weian Zeng, Lanjun Zhang

**Affiliations:** ^1^State Key Laboratory of Oncology in South China, Collaborative Innovation Center for Cancer Medicine, Guangzhou, China; ^2^Department of Thoracic Surgery, Sun Yat-sen University Cancer Center, Guangzhou, China; ^3^Department of Thoracic Surgery, National Cancer Center/National Clinical Research Center for Cancer/Cancer Hospital and Shenzhen Hospital, Chinese Academy of Medical Sciences and Peking Union Medical College, Shenzhen, China; ^4^Department of Anesthesiology, Huizhou Municipal Central Hospital, Huizhou, China; ^5^Department of Anesthesiology, Sun Yat-sen University Cancer Center, Guangzhou, China; ^6^Department of Thoracic Surgery, The First Affiliated Hospital, School of Medicine, Zhejiang University, Hangzhou, China

**Keywords:** lung adenocarcinoma (LUAD), GILncSig, genome instability, TCGA, TP53

## Abstract

**Background:**

Genomic instability is one of the representative features of cancer evolution. Recent research has revealed that long noncoding RNAs (lncRNAs) play a critical role in maintaining genomic instability. Our work proposed a gene signature (GILncSig) based on genomic instability-derived lncRNAs to probe the possibility of lncRNA signatures as an index of genomic instability, providing a potential new approach to identify genomic instability-related cancer biomarkers.

**Methods:**

Lung adenocarcinoma (LUAD) gene expression data from an RNA-seq FPKM dataset, somatic mutation information and relevant clinical materials were downloaded from The Cancer Genome Atlas (TCGA). A prognostic model consisting of genomic instability-related lncRNAs was constructed, termed GILncSig, to calculate the risk score. We validated GILncSig using data from the Gene Expression Omnibus (GEO) database. In this study, we used R software for data analysis.

**Results:**

Through univariate and multivariate Cox regression analyses, five genomic instability-associated lncRNAs (*LINC01671, LINC01116, LINC01214, lncRNA PTCSC3*, and *LINC02555*) were identified. We constructed a lncRNA signature (GILncSig) related to genomic instability. LUAD patients were classified into two risk groups by GILncSig. The results showed that the survival rate of LUAD patients in the low-risk group was higher than that of those in the high-risk group. Then, we verified GILncSig in the GEO database. GILncSig was associated with the genomic mutation rate of LUAD. We also used GILncSig to divide TP53 mutant-type patients and TP53 wild-type patients into two groups and performed prognostic analysis. The results suggested that compared with TP53 mutation status, GILncSig may have better prognostic significance.

**Conclusions:**

By combining the lncRNA expression profiles associated with somatic mutations and the corresponding clinical characteristics of LUAD, a lncRNA signature (GILncSig) related to genomic instability was established.

## Introduction

According to the latest data released by the International Agency for Research on Cancer (IARC) under the World Health Organization (WHO), there were 2.2 million new cases of lung cancer, accounting for 11.6% of all new cancers. Among all cancer types, lung cancer still has the highest global mortality rate. In China, the mortality and morbidity of lung cancer are ranked first among cancers. According to statistics, non-small cell lung cancer (NSCLC), which mainly includes lung adenocarcinoma (LUAD) and lung squamous cell carcinoma (LUSC), accounts for approximately 85% of lung cancer cases (1). LUAD is the most common subtype of NSCLC, accounting for approximately 40% of all lung cancer cases (2). At present, the indicators used to predict the prognosis and monitor the recurrence of lung cancer patients mainly include age, tumor grade, lymph node involvement, driver gene mutation, etc. Although great improvements have been made in recent years in screening, diagnosis and treatment, effective molecular biomarkers for predicting the biological behavior of NSCLC are still lacking due to the complexity of the pathogenesis and progression mechanisms. Therefore, to provide better prognostic indicators for NSCLC patients, the identification of novel and more effective molecular biomarkers is necessary.

In recent years, several studies have reported that genomic instability is one of the evolving hallmarks of cancers (3, 4). Moreover, genomic instability is a significant prognostic risk factor. The accumulation of genomic instability is regarded as a critical feature of tumorigenesis and is associated with tumor progression and survival (5). For example, David et al. demonstrated that in clear cell renal cell carcinoma (ccRCC), genetic instability is enriched in high-grade tumors and is associated with poor prognosis (6). It has been reported that germline mutations in BRCA1/2 cause genomic instability, which predisposes individuals to breast cancer (7). Vinayak et al. found that many tumors are characterized by increased genomic instability due to hypoxia adaptation, which is associated with poor clinical prognosis (8). Genomic instability can lead to the frequent recurrence of multiple myeloma and susceptibility to the formation of therapeutic resistance (9).

Long noncoding RNAs (lncRNAs) are a kind of noncoding RNA with a transcript length of more than 200 bases. During the past decade, lncRNAs have been proven to play an important role in the regulation of RNA metabolism, transcription, translation and apoptosis. Moreover, the aberrant expression of lncRNAs has been implicated in the occurrence and progression of cancer (10). The abnormal expression of numerous lncRNAs in various cancers has been reported in many studies; however, the specific function of these lncRNAs remains largely unknown (11–14). Previous studies have revealed that lncRNAs play a key role in maintaining genomic instability (15). For instance, studies have confirmed that the activation of LIN28B in LUAD can interfere with DNA damage repair and affect the cell cycle and genomic instability, thus promoting the proliferation and metastasis of tumor cells (16). Hu et al. showed that cells depleted of BGL3 exhibit genomic instability, making them prone to DNA damage (17). A recent study reported that a particular lncRNA termed NORAD (*noncoding RNA activated by DNA damage*) was related to genomic instability (15). Although a few lncRNAs have been shown to be associated with genomic instability, the clinical role of genomic instability-related lncRNAs in cancers remains unclear.

In our study, we combined the expression profiles of lncRNAs with the somatic mutation profiles in the lung cancer genome to establish a predictive model composed of lncRNAs to explore the value of lncRNA signatures as an indicator of genomic instability to improve their clinical predictive utility.

## Method

### Data Acquisition and Processing

We downloaded LUAD gene expression data from a level-3 RNA sequencing (RNA-seq) fragments per kilobase of transcript per million mapped reads (FPKM) dataset as well as somatic mutation information and the corresponding clinical information from The Cancer Genome Atlas (TCGA) database. There were 19,322 mRNAs and 13,162 lncRNAs acquired *via* annotation. The “maftools” package was used to analyze and sum the somatic mutation profiles (18). The clinical information of LUAD patients in the TCGA cohort, including sex, age, pathologic stage, pathologic TNM stage, survival time, and survival state, was collected and used for the subsequent analyses. All of the patients included in this study were divided into a training set and a testing set. The GSE50081 dataset from the Gene Expression Omnibus (GEO) database was used as an external validation cohort. We present a concise summary of the pathological and clinical traits in **Table 1**.

**Table 1:** Clinical information for three LUAD patients sets in this study.

Covariates	Type	Train	Test	Total	Pvalue
age	<=65	108 (48.87%)	105 (48.17%)	213 (48.52%)	0.9585
age	>65	113 (51.13%)	113 (51.83%)	226 (51.48%)	
gender	FEMALE	114 (51.58%)	127 (58.26%)	241 (54.9%)	0.1905
gender	MALE	107 (48.42%)	91 (41.74%)	198 (45.1%)	
stage	Stage I-II	177 (80.09%)	169 (77.52%)	346 (78.82%)	0.5882
stage	Stage III-IV	44 (19.91%)	49 (22.48%)	93 (21.18%)	
T	T1-2	196 (88.69%)	189 (86.7%)	385 (87.7%)	0.6244
T	T3-4	25 (11.31%)	29 (13.3%)	54 (12.3%)	
M	M0	212 (95.93%)	207 (94.95%)	419 (95.44%)	0.7947
M	M1	9 (4.07%)	11 (5.05%)	20 (4.56%)	
N	N0	142 (64.25%)	149 (68.35%)	291 (66.29%)	0.4199
N	N1-3	79 (35.75%)	69 (31.65%)	148 (33.71%)	

A total of 38 clinical samples were collected at Sun Yat-sen University Cancer Center during surgery from October 2020 to March 2021. This study was conducted based on the Declaration of Helsinki, and was approved by the Ethics Committee of Sun Yat-sen University Cancer Center.

### Identification of LncRNAs Related to Genomic Instability

To identify lncRNAs related to genomic instability, we combined the expression profiles of lncRNAs in tumor genomes with somatic mutation profiles. First, we calculated the accumulated amounts of somatic mutations in every patient and ranked the patients in descending order. The top 25% of patients with frequent somatic mutations were designated the genomic instability (GU)-like group, and the bottom 25% were designated the genomic stability (GS)-like group. Then, the differential expression of lncRNAs was analyzed with the Wilcoxon test by the “limma” package of R software. The thresholds were set as |log2 fold change (FC)| > 1 and adjusted false discovery rate (FDR) < 0.01, with an adjusted P < 0.05, and the differentially expressed lncRNAs were considered related to genomic instability.

### Construction of a Coexpression Network of LncRNAs and mRNAs and Prediction of the Functions of LncRNAs Through Bioinformatics Analysis

The “igraph” package was used to construct a lncRNA−mRNA coexpression network. To identify the biological functions of the lncRNAs, the “clusterProfiler” package was applied to perform Kyoto Encyclopedia of Genes and Genomes (KEGG) and Gene Ontology (GO) analyses. An FDR (q value) <0.05 was considered the statistical standard.

### Construction of the Risk Model for Prognostic Prediction

The “survival” package was employed to perform univariate and multivariate Cox regression analyses to assess the relationship between the expression levels of lncRNAs related to genomic instability and the prognosis of LUAD patients. On the basis of the multivariate Cox regression analysis, a prognostic model consisting of genomic instability-associated lncRNAs was constructed, which we termed GILncSig, to calculate the risk score. The GILncSig of each LUAD patient was determined by the following equation:


$$\begin{array}{c} \text{GILncSig}={\text{Exp (lncRNA}}_{1})\times {\text{coef (lncRNA}}_{1})\\ +{\text{Exp (lncRNA}}_{2})\times {\text{coef (lncRNA}}_{2})\;\;\ldots \\ \ldots +{\text{Exp (lncRNA}}_{\text{i}})\times {\text{coef (lncRNA}}_{\text{i}}) \end{array}$$

### Quantitative Real−Time PCR Analysis

The total RNA of 38 clinical samples was extracted using TRIzol reagent (Invitrogen, Cat. #15596) and reverse-transcribed using PrimeScript RT reagent Kit with gDNA Eraser (TaKaRa, Cat. #DRR047A). Quantitative real−time PCR (qRT-PCR) was used to verify the expression of genes of the GILncSig in LUAD tissues. We used Bio-Rad instrument, and applied SYBR Green (ES Science) for real-time quantitative PCR. The expression levels were calculated using the 2^ΔΔCT^ method and normalized to β-actin. The primer sequences related to this study are shown in **Table S3**.

### Statistical Analysis

We used Kaplan-Meier analysis to construct a survival curve, and the log-rank test was used to assess the significance of the differences. Multivariate Cox regression analysis was applied to evaluate the independence of GILncSig from other clinical factors. Significance was defined as P < 0.05. We applied the time-dependent receiver operating characteristic (ROC) curve to appraise the predictive capability of GILncSig. The statistical analysis was implemented with R software v4.0.3.

## Results

### Identification of Genomic Instability-Associated LncRNAs in Lung Cancer

**Figure 1:**
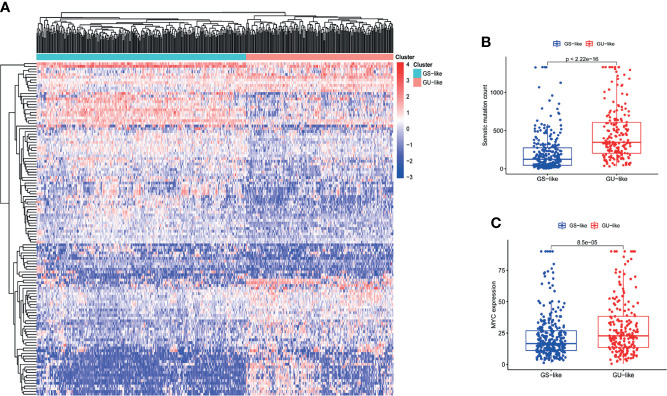
The recognition of genomic instability-associated lncRNAs in patients with LUAD. **(A)** On the basis of the expression patterns of 123 candidate lncRNAs associated with genomic instability, unsupervised clustering was performed in 497 LUAD patients. The blue cluster on the left represents the GS-like group, the red cluster on the right represents the GU-like group. **(B)** Boxplots of somatic mutations in the GS-like group and GU-like group. The accumulative mutation count of somatic cells in the GS-like group is dramatically lower than that in the GU-like group. **(C)** Boxplots of MYC expression level in the GS-like group and GU-like group. The expression level of MYC in the GS-like group is dramatically lower than that in the GS-like group.

We acquired a gene matrix and corresponding clinical information from the TCGA database, including 497 tumor tissues and 54 normal tissues. *Via* annotation, we detected 19,322 mRNAs and 13,162 lncRNAs. To identify lncRNAs related to genomic instability, we calculated the accumulated amounts of somatic mutations of each patient and ranked them in decreasing order. Based on the number of accumulated somatic mutations of the patients, the top 25% (n= 130) and the bottom 25% (n= 125) were allotted to the GU-like and GS-like groups, respectively. The “limma” package of R software was used to analyze the differential expression of all patient samples, and in comparing the lncRNA expression profiles of the 130 samples in the GU-like group and 125 samples in the GS-like group, it was found that there were dramatic differences between the two groups. A total of 123 lncRNAs were identified as significantly differentially expressed (log FC threshold >1 and FDR threshold <0.01, **Table S1**). With the 123 differentially expressed lncRNAs, the “limma” and “sparcl” packages of R software were applied to perform unsupervised hierarchical clustering analysis on 551 patients from the TCGA dataset. According to the expression levels of the 123 differentially expressed lncRNAs, all 551 patients were classified into two groups (**Figure 1A**). The somatic mutation count was significantly different between the GU-like group and the GS-like group (**Figure 1B**). Compared with the GS-like group, the accumulated somatic mutations in the GU-like group were dramatically higher. Next, we compared the expression level of the MYC gene (a driver gene associated with genomic instability) (19, 20) between the two groups. As shown in **Figure 1C**, the GU-like group had a dramatically higher expression of MYC than the GS-like group.

#### Construction of a Genomic Instability-Associated LncRNA Signature Model

To further investigate the prognostic roles of these lncRNAs in genomic instability, 497 LUAD patients from the TCGA database were randomly classified into a training set and testing set using R software. To identify prognostic-associated lncRNAs, we carried out univariate Cox regression analysis to analyze the correlation between the expression levels of the 123 genomic instability-related lncRNAs and overall survival (OS) in the training set. There were 8 genomic instability-associated lncRNAs that were significantly correlated with the prognosis of patients with LUAD (P <0.05, **Table S2**). Furthermore, we performed multivariate Cox analysis on these 8 candidate lncRNAs to screen out independent prognostic lncRNAs. Finally, 5 candidate lncRNAs derived from the 8 lncRNAs mentioned above were identified as independent prognostic lncRNAs by multivariate Cox analysis (P <0.05, **Table 2**). To assess the prognostic risk of LUAD patients, we established a lncRNA signature (GILncSig) related to genomic instability based on the multivariate Cox analysis coefficients and expression levels of the 5 lncRNAs that were independent prognostic factors for genomic instability. The GILncSig score was calculated by the following formula:


$$\begin{array}{c} \text{GILncSig}=(-0.08497\times \text{Exp PTCSC}3)+(-0.26220\times \text{Exp LINC}02555)+(0.04078\times \text{Exp LINC}01671)\\ +(0.09852\times \text{Exp LINC}01116)+(0.06491\times >\text{Exp LINC}01214) \end{array}$$

**Table 2:** Five prognosis-related genome instability-associated lncRNAs by multivariate Cox regression analysis.

LncRNA	coef	HR	HR.95L	HR.95H	p-value
PTCSC3	-0.08497	0.918544	0.828557	1.018303	0.106273
LINC02555	-0.2622	0.76936	0.559437	1.058056	0.106782
LINC01671	0.040781	1.041624	1.014932	1.069017	0.002077
LINC01116	0.098516	1.103532	0.996312	1.222292	0.058877
LINC01214	0.06491	1.067063	1.006868	1.130858	0.028452

**Figure 2:**
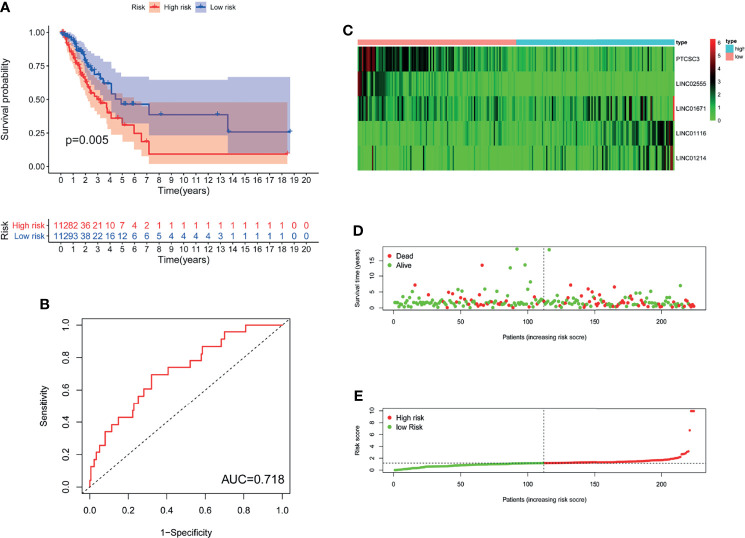
The genomic instability-derived lncRNA signature (GILncSig) related to overall survival (OS) of LUAD in training set. **(A)** Log-rank test was used to draw a Kaplan Meier survival curve to predict the overall survival of GILncSig in low-risk or high-risk patients in training set. **(B)** Receiver Operating Characteristic (ROC) analysis of GILncSig in training set. **(C)** Heat map of 5 genomic instability-derived lncRNA expression patterns in training set. **(D)** The survival status of LUAD patients. The dotted line represents the median of the risk score; the number of deaths in the right part of the patient is greater than the left part, indicating that as the risk score increases, the risk of death increases. **(E)** Risk score distribution of LUAD patients.

In GILncSig, the coefficients of LINC01671, LINC01116, and LINC01214 were positive, suggesting that they may be risk factors since their high expression was related to poor prognosis, whereas the lncRNAs PTCSC3 and LINC02555 were protective factors, and their high expression was related to good prognosis. GILncSig was used to calculate the risk score for each patient in the training set, and then the median risk score (1.176) was used as a threshold to divide the patients into a high-risk group and a low-risk group. Patients whose scores were lower than the threshold were assigned to the low-risk group; otherwise, they were assigned to the high-risk group. **Table 3** sums up the relevance between GILncSig risk score and various clinicopathological parameters in LUAD patients. Among them, patient survival status, TNM stage, TMB and N stage were dramatically related to GILncSig risk score. Kaplan-Meier analysis demonstrated that the OS of the high-risk group was markedly poorer than that of the low-risk group (P <0.05; **Figure 2A**). We further conducted time-dependent ROC analysis to assess the predictive power of GILncSig. The results showed that the area under the ROC curve (AUC) of GILncSig was 0.718 for the training cohort (**Figure 2B**). We classified the samples in the training set based on the scores, and the expression level of each lncRNA in the GILncSig model, the status of patient survival, and the risk score distribution are outlined in **Figures 2C–E**. With regard to high-score patients, LINC01671, LINC01116, and LINC01214 were considered risk lncRNAs, and their expression levels were upregulated, while PTCSC3 and LINC02555 were considered protective lncRNAs, and their expression levels were downregulated. Moreover, GILncSig showed opposite expression patterns in low-score patients compared with high-score patients. In addition, there were more deaths among patients with high-risk scores than among those with low-risk scores.

**Table 3:** Correlations between GILncSig and clinical characteristics of LUAD patients in TCGA.

Characteristics	No. of patients	GILncSig		*P*-value
		Low (n = 204)	High (n = 230)	
**Age (y)**				
≤65	211 (48.6%)	110 (47.8%)	101 (49.5%)	0.726
>65	223 (51.4%)	120 (52.2%)	103 (50.5%)	
**Gender**				
Female	238 (54.8%)	127 (55.2%)	111 (54.4%)	0.866
male	196 (45.2%)	103 (44.8%)	93 (45.6%)	
**T stage**				
T1-2	381 (87.8%)	199 (86.5%)	182 (89.2%)	0.392
T3-4	53 (12.2%)	31 (13.5%)	22 (10.8%)	
**N stage**				
N0	287 (66.1%)	138 (60%)	149 (73%)	**0.004**
N1-3	147 (33.9%)	92 (40%)	55 (27%)	
**M stage**				
M0	414 (95.4%)	216 (93.9%)	198 (97.1%)	0.119
M1	20 (4.6%)	14 (6.1%)	6 (2.9%)	
**TNM stage**				
I-II	342 (78.8%)	168 (73%)	174 (85.3%)	**0.002**
III-IV	92 (21.2%)	62 (27%)	30 (14.7%)	
**TMB**				
High	93 (21.4%)	62 (27%)	31 (15.2%)	**0.003**
Low	341 (78.6%)	168 (73%)	173 (84.8%)	
**Status**				
Alive	286 (65.9%)	137 (59.6%)	149 (73%)	**0.003**
Dead	148 (34.1%)	93 (40.4%)	55 (27%)	

The bold number means statistical significance.

#### Independent Validation of GILncSig in the LUAD TCGA Dataset

**Figure 3:**
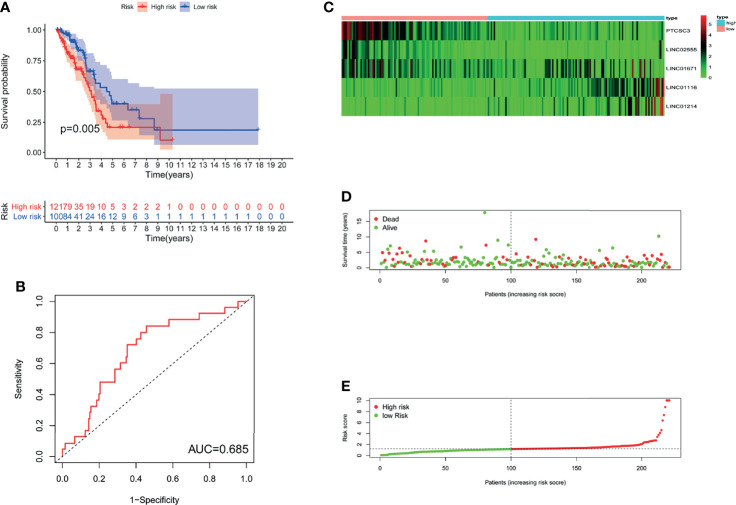
The GILncSig associated with OS of LUAD in testing set. **(A)** Log-rank test was performed to draw a Kaplan Meier survival curve to predict the overall survival of GILncSig in low-risk or high-risk patients in testing set. **(B)** ROC analysis of the GILncSig in testing set. ***(*C*)*** Heat map of 5 genomic instability-derived lncRNA expression patterns in testing set. ***(*D*)*** The survival status of LUAD patients. The dotted line represents the median of the risk score; the number of deaths in the right part of the patient is greater than the left part, indicating that as the risk score increases, the risk of death increases. **(E)** Risk score distribution of LUAD patients.

To investigate the stability of GILncSig, we tested the prognostic value of GILncSig in the TCGA testing set (n=221). Using the same GILncSig that was established based on the training set, we divided the 221 patients in the testing set into a high-risk group (n= 121) and a low-risk group (n= 100). The results of survival analysis showed that OS was significantly different between the two groups. As shown in **Figure 3A**, compared with patients in the high-risk group, patients in the low-risk group had a better OS rate (P= 0.005). Then, we performed time-dependent ROC analysis to appraise the predictive power of GILncSig in the testing set, and the AUC of GILncSig was 0.685 for the testing cohort (**Figure 3B**). The GILncSig expression, patient survival status and risk score distribution in the testing set are shown in **Figures 3C–E**.

#### Further Validation of GILncSig Using All Patients From the TCGA Cohort

**Figure 4:**
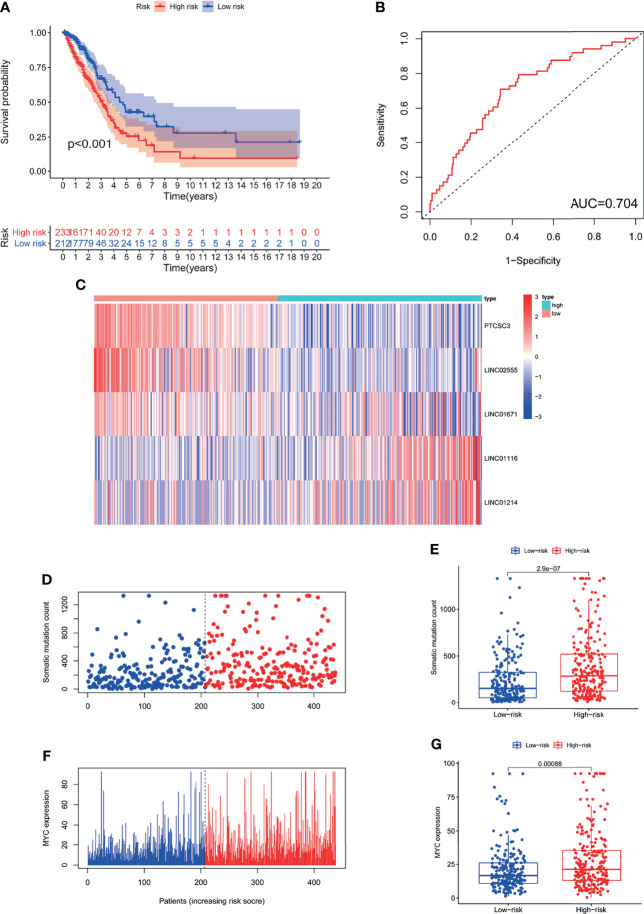
The GILncSig associated with OS of LUAD and property assessment of the GILncSig in the TCGA set. **(A)** Log-rank test was performed to draw a Kaplan Meier survival curve to predict the overall survival of GILncSig in low- or high-risk patients in the TCGA set. **(B)** ROC analysis of GILncSig in the TCGA set. **(C)** Heat map of 5 genomic instability-derived lncRNA expression patterns in TCGA set. **(D)** The somatic mutation count distribution in the TCGA set. **(E)** Comparison analysis of somatic mutation count for patients in low- and high-risk groups in the TCGA set. **(F)** The distribution of MYC expression in the TCGA set. **(G)** Comparison analysis of MYC expression in low- and high-risk groups in the TCGA set.

We assessed the prognostic value of GILncSig in the TCGA cohort. Patients in the TCGA cohort were similarly divided into two groups: a high-risk group containing 233 patients and a low-risk group containing 212 patients. The OS of patients in the low-risk group was significantly better than that of patients in the high-risk group (P<0.001; **Figure 4A**). The ROC curve was used to analyze the TCGA dataset, and the results obtained were in line with the above results (AUC = 0.704; **Figure 4B**). The GILncSig expression, somatic mutation count distribution and MYC expression in the TCGA set are shown in **Figures 4C, D, F**. As shown in **Figure 4E**, there was a significant difference in the somatic mutation count distribution between the low-risk group and the high-risk group (P = 2.9e−07). Additionally, the MYC expression level in the low-risk group was markedly lower than that in the high-risk group (P= 0.00031; **Figure 4G**).

#### Construction of a LncRNA−mRNA Co-Expression Network and LncRNA Functional Predictions

**Figure 5:**
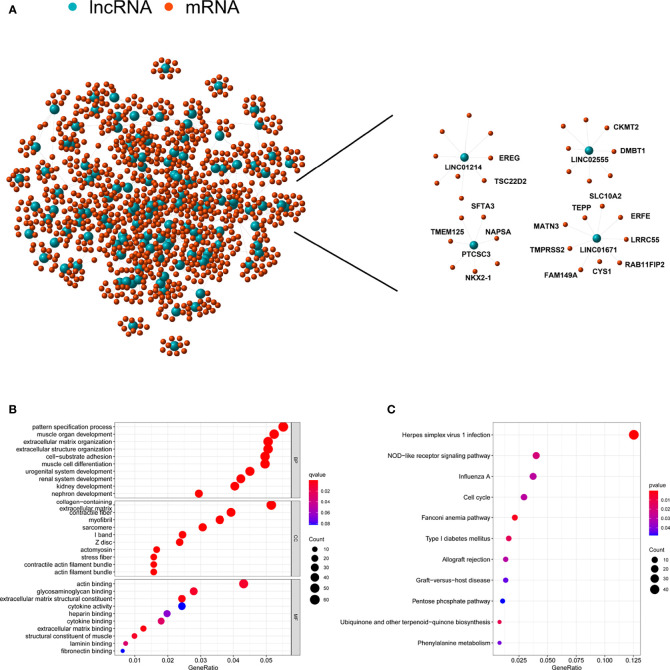
Functional annotation of lncRNA related to genomic instability and construction of lncRNA-mRNA co-expression network. **(A)** Co-expression network of genomic instability-associated lncRNAs and mRNAs. LncRNAs were represented by the blue circles, and mRNAs were represented by the red circles. **(B)** Gene Ontology (GO) enrichment analysis for mRNAs co-expressed lncRNAs. **(C)** Kyoto Encyclopedia of Genes and Genomes (KEGG) pathway analysis for mRNAs co-expressed lncRNAs.

To probe the potential biological functions of the 123 genomic instability-associated lncRNAs, a co-expression network was constructed between mRNAs and the 123 lncRNAs that were differentially expressed. The nodes that compose this co-expression network represent lncRNAs and mRNAs, and related lncRNAs and mRNAs are linked together. The genes associated with cancer prognosis related to our GILncSig are highlighted on the right of **Figure 5A**. Subsequently, we implemented GO enrichment analysis to predict the potential functions of these genes. GO analysis showed that in terms of biological processes (BPs), these genes might be involved in cell-substrate adhesion, muscle organ development, extracellular structure organization, and pattern specification processes. In terms of the cellular components (CCs), extracellular matrix, collagen-containing, contractile fiber and myofibrils were prominently enriched. In the molecular function (MF) analysis, these genes were remarkably enriched in actin binding, glycosaminoglycan binding, extracellular matrix structural constituent and extracellular matrix binding (**Figure 5B**). The KEGG pathway analysis showed that these genes were significantly enriched in 11 pathways, including herpes simplex virus 1 infection, NOD-like receptor signaling pathway, ubiquinone and other terpenoid-quinone biosynthesis, and the cell cycle (**Figure 5C**).

### Independent Prognostic Analysis of GILncSig

**Figure 6:**
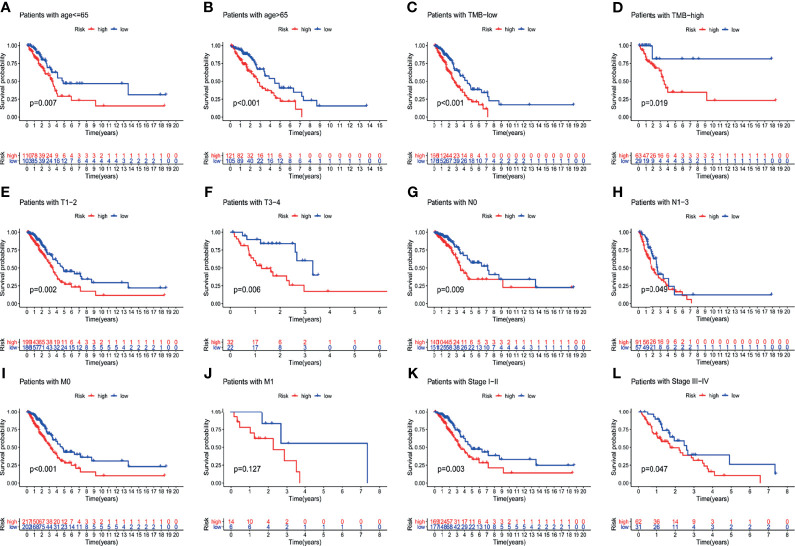
Explore the performance of GILncSig in the overall survival of various clinical factors in LUAD patients by using the Kaplan-Meier survival curve. Groups classified by age **(A, B)**; TMB **(C, D)**; tissue involvement **(E, F)**; lymphatic involvement **(G, H)**; metastasis **(I, J)** and stages **(K, L)**.

To verify the relationship between the prognostic prediction model GILncSig and various pathological factors of patients, Chi-squared tests were used to compare the GILncSig and the clinicopathological data of LUAD patients (**Table 3**). To appraise the independent prognostic value of GILncSig from various clinical factors, we performed multivariate Cox analysis including age, sex, T stage, N stage, M stage and the GILncSig-based prognostic model. The multivariate analysis results showed a significant association between GILncSig and OS in each set after adjustment for age, sex, T stage, N stage and M stage (**Table 4**). All patients with LUAD in the TCGA cohort were divided into two groups based on their age: the over 65 years old group and the under 65 years old group (including patients 65 years old). Compared with older patients (**Figure 6B**), the OS of the high-risk group and the low-risk group was significantly different in the young patients (**Figure 6A**). Next, all lung cancer patients were divided into two groups by tumor mutational burden (TMB), T stage, N stage, and M stage. According to TMB expression and pathologic stage, the patients in each group could be further classified into high- and low-risk groups. Similarly, the OS of patients in the low-risk group was better than that of patients in the high-risk group.

**Table 4:** Univariate and Multivariate Cox regression analysis of the GILncSig and overall survival in different patient sets.

Variables	Univariable model			Multivariable model		
	HR	95% CI	p-value	HR	95% CI	p-value
Training set						
age	1.01639	0.99105-1.04238	0.206967			
gender	0.971905	0.61358-1.53948	0.903347			
T	1.282301	0.94688-1.73655	0.10802			
N	1.512145	1.16412-1.96421	0.001944	1.448928	1.10816-1.89449	0.006714
M	1.726692	0.62629-4.76052	0.291146			
GILncSig	1.182901	1.11023-1.26033	2.08E-07	1.168063	1.09414-1.24699	3.21E-06
Testing set						
age	1.00906	0.98679-1.03183	0.428217			
gender	1.288724	0.82256-2.01907	0.268176			
T	1.883562	1.42049-2.49759	1.09E-05	1.518812	1.11782-2.06364	0.007537
N	2.107405	1.62118-2.73946	2.55E-08	1.862951	1.41147-2.45884	1.11E-05
M	1.92918	0.92450-4.02569	0.079982			
GILncSig	1.097863	1.04963-1.14831	4.64E-05	1.060405	1.01205-1.11107	0.013783
TCGA set						
age	1.012345	0.99562-1.02935	0.148942			
gender	1.119877	0.81228-1.54396	0.48956			
T	1.533885	1.25392-1.87636	3.17E-05	1.204072	0.97288-1.49021	0.087785
N	1.7257	1.44021-2.06778	3.35E-09	1.578085	1.29653-1.92078	5.36E-06
M	1.848907	1.02319-3.34098	0.04176	1.577883	0.86367-2.88272	0.137995
GILncSig	1.114489	1.07825-1.15195	1.30E-10	1.09146	1.05451-1.12971	6.35E-07

### Comparison of the Predictive Ability of GILncSig and TP53 Mutation Status

**Figure 7:**
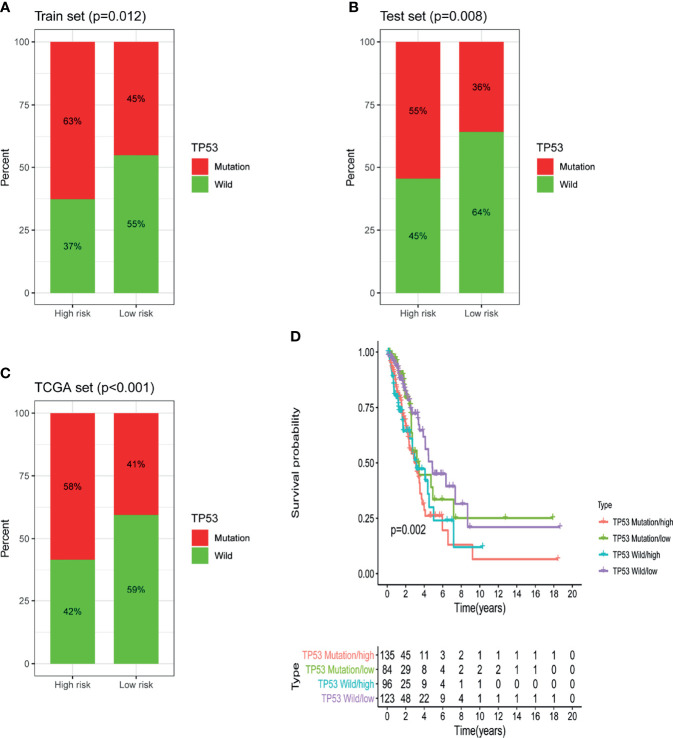
Identification of the relationship between TP53 somatic mutation and GILncSig. **(A)** Comparison of the mutation ratio of TP53 in the high-risk and low-risk groups in the training set. **(B)** Comparison of the mutation ratio of TP53 in the high-risk and low-risk groups in the testing set. **(C)** Comparison of the mutation ratio of TP53 in the high-risk and low-risk groups in the TCGA set. **(D)** Survival analysis of LUAD patients was categorized on the basis of the GILncSig and the TP53 mutation status.

Further analysis showed that in the training set, testing set and TCGA set, the proportion of patients with TP53 mutations in the low-risk group was notably lower than that in the high-risk group. In the training dataset, 63% of patients in the high-risk group had TP53 mutations and 45% of patients in the low-risk group had TP53 mutations; the percentage of patients with TP53 mutations was significantly lower in the low-risk group than in the high-risk group (P = 0.012; **Figure 7A**). The same conclusion was made in the testing dataset and the TCGA dataset. In the testing dataset, 36% of patients in the low-risk group had TP53 mutations, which was notably lower than the 55% of patients in the high-risk group who had TP53 mutations (P = 0.008; **Figure 7B**). Similarly, in the TCGA set, 41% of patients in the low-risk group had TP53 mutations, which was dramatically lower than the 58% of patients in the high-risk group who had TP53 mutations (P <0.001; **Figure 7C**). The above results suggested that GILncSig might be related to the mutation status of TP53. Studies have found that tumors with TP53 comutations had a higher somatic mutation burden and a higher degree of copy number genomic instability, and TP53 comutation was a negative prognostic marker in epidermal growth factor receptor (EGFR)-mutant LUAD and a predictor of poor clinical effect of EGFR-tyrosine kinase inhibitor (TKI) treatment (21). Hence, we further verified whether GILncSig had better predictive power than TP53 mutation status. According to the survival rates, we used GILncSig to classify patients with wild-type and mutant TP53 sequences into high-risk groups and low-risk groups and obtained 4 risk groups. Patients with wild-type TP53 in the low-risk group were defined as the TP53 wild-type/low group, and patients with wild-type TP53 in the high-risk group were defined as the TP53 wild-type/high group. Similarly, patients with TP53 mutation in the low-risk group were defined as the TP53 mutation/low group, and patients with TP53 mutation in the high-risk group were defined as the TP53 mutation/high group. As shown in **Figure 7D**, the TP53 wild-type/low group had better outcomes than the TP53 wild-type/high group; similarly, the TP53 mutation/low group had better outcomes than the TP53 mutation/high group. However, patients in the TP53 wild-type/low group had similar outcomes to those in the TP53 mutation/low group. The same situation occurred in the other two groups. Nevertheless, the survival curve of the TP53 wild-type/high group was lower than that of the TP53 mutation/low group. These results imply that compared with TP53 mutation status, GILncSig has more advantages in predicting prognosis. We then used the same method to verify the relationship between GILncSig and lung cancer driver genes (EGFR and ALK) and obtained similar conclusions (**Figures S1A, B**).

### Establish a Nomogram Based on GILncSig and a Variety of Clinical Factors

**Figure 8:**
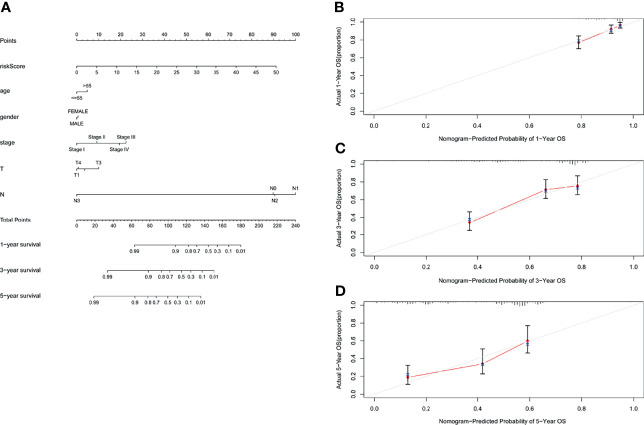
Nomogram and calibration charts of LUAD patients based on the GILncSig. **(A)** Nomogram for predicting LUAD prognosis. **(B)** Calibration plots for the 1- year nomogram. **(C)** Calibration plots for the 3- year nomogram. **(D)** Calibration plots for the 5- year nomogram.

In order to evaluate the role of GILncSig in predicting the prognosis of patients with lung adenocarcinoma, we used “rms” package of R software to establish a nomogram chart based on the results of the multivariate Cox analysis to predict the 1-year, 3-year, and 5-year OS of LUAD patients on the basis of various clinical factors and riskscore (**Figure 8A**). The calibration plots of nomogram show that the probability of using nomogram to predict OS in 1-, 3- and 5- years is in good agreement with actual observations (**Figures 8B–D***)*.

### Further Validation of GILncSig in the GEO Database

**Figure 9:**
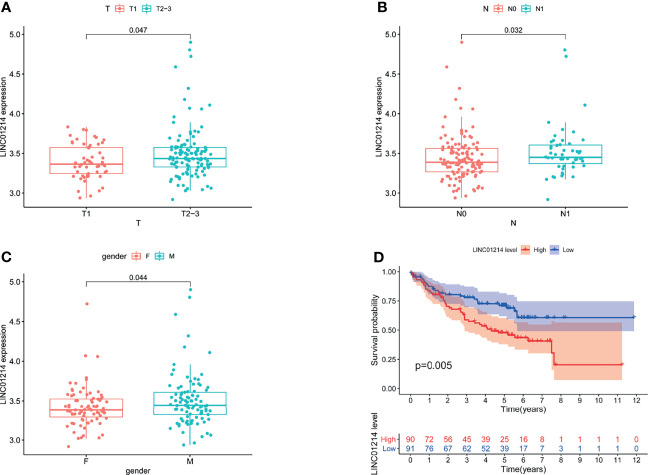
Performance evaluation of LINC01214 in GSE50081 dataset. **(A)** Boxplots for LINC01214 expression among patients with different T stage (tumor size) in GSE50081 dataset. **(B)** Boxplots for LINC01214 expression among patients with different N stage (lymph node metastasis) in GSE50081 dataset. **(C)** Boxplots for LINC01214 expression among patients with different gender in GSE50081 dataset. **(D)** Kaplan Meier curve analysis was used to verify the overall survival rate of LINC01214 expression level in GEO database in different groups based on GILncSig.

**Figure 10:**
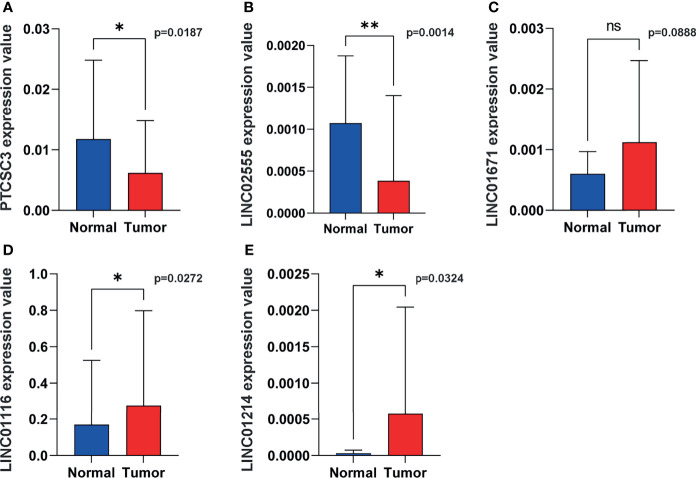
Representative lncRNAs expression in tumor and normal tissues. **(A)** PTCSC3; **(B)** LINC02555; **(C)**, LINC01671; **(D)** LINC01116; **(E)** LINC01214. *P < 0.05; **P < 0.01; ns, P < 0.05.

To verify whether GILncSig has the same prognostic performance in other datasets, we used the GEO database for verification. Of the five lncRNAs that constitute GILncSig, we found only one (LINC01214) in the GSE50081 dataset. Therefore, we explored the correlation of LINC01214 with LUAD and genomic instability in the GSE50081 dataset from the GEO database. We found that in the GSE50081 dataset, the expression of LINC01214 had a good correlation with T stage (tumor size). As shown in **Figure 9A**, there was a significant difference in the expression level of LINC01214 among patients with different T stages (P = 0.047). Likewise, patients without lymph node metastasis were more likely to have low LINC01214 expression than those with lymph node metastasis (P = 0.032; **Figure 9B**). In addition, the expression level of LINC01214 was significantly different in patients of different sexes (P = 0.044; **Figure 9C**). As shown in **Figure 10D**, the low LINC01214 expression group had a better prognosis than the high LINC01214 expression group in the GSE50081 dataset.

### Validation of Clinical Samples

In order to verify the expression of genetic instability-related molecules in GILncSig in lung cancer and normal tissues, we performed qRT - PCR analysis on clinical samples. Consistent with our gene signature, the results showed that the expression level of high risk lncRNAs (LINC01214, LINC01116 and LINC01671) were lower in normal lung tissues compared to cancer tissues, indicating they may be associated with tumor pathogenesis. However, the expression level of LINC02555 and PTCSC3 were higher in normal lung tissues, reflecting they may serve as repressive roles in tumor growth or progression (**Figure 10**).

## Discussion

Despite advances in our understanding of the risk, diagnosis, and treatment options for lung cancer, it still accounts for 11.6% and 18.4% of global cancer morbidity and mortality, respectively (22). The survival time of lung cancer patients is disappointing, especially for patients with high risk (23). Biomarkers and models that accurately predict lung cancer risk may aid in detecting people at higher risk who should undergo intensive interventions (24). Unfortunately, many challenges exist with this strategy, and there is an unmet need for more reliable prediction models.

lncRNAs [which are more than 200 nucleotides in length (25)] are involved in various cellular processes *via* multiple different mechanisms (26). It has been proven that lncRNAs regulate human cancer progression, as well as the response to therapy (27). In this regard, lncRNAs should be superior biomarkers for predicting cancer prognosis. In lung cancer, the lncRNA HOTAIR is involved in cigarette smoke extract (CSE)-induced carcinogenesis (28), and LINC01186 inhibits cancer migration and invasion through epithelial-mesenchymal transition (29). Additionally, high lncRNA SBF2-AS1 expression promotes proliferation in lung cancer (30). Recently, some researchers identified a 4-lncRNA signature to predict the prognosis of NSCLC patients (31); however, the clinical application of prognostic lncRNA models remains limited. Genetic instability plays a crucial role in lung cancer progression, promoting tumor survival and proliferation (32), as well as lung cancer evolution (33). Because lung cancer has a high mutational burden (34) and certain TKIs have been applied in patients with somatic mutations in EGFR or translocations involving ALK or ROS1 (35), genetic instability should be a sensitive risk predictor. However, prediction models based on genome-wide genomic instability-associated lncRNAs in lung cancers are still rare. Thus, we established a model based on genomic instability-associated lncRNA expression to predict the clinical outcomes of patients.

We calculated the cumulative amounts of somatic mutations in every patient and ranked them in order and then divided the top 25% and the bottom 25% of the patients into two groups. Higher cumulative somatic mutations and MYC expression were found in the GU-like group. High MYC expression has been proven to be associated with tumor progression and poor survival (36), and the MYC status determines the tumor immunophenotype (37) in lung cancer. Next, candidate lncRNAs were chosen from the differentially expressed lncRNAs between the two groups, and then we performed multivariate Cox analysis including the five candidate lncRNAs. The patients were classified into a high-risk group and a low-risk group based on the expression levels of the candidate lncRNAs. Consistent with previous findings, patients in the high-risk group had higher cumulative somatic mutations and MYC expression, as well as shorter survival times. LINC01116 was shown to enhance the expression of MYC protein by inducing its translation. Further, the effect of LINC01116 deletion could be partially restored by upregulation MYC expression (38). However, overexpression of PTCSC3 would suppress the expression of MYC through targeting LRP6 (39). Those two lncRNAs in our model also showed opposite effect on regulating STAT3. It has been reported that LINC01116 is overexpressed in lung cancer and that it could upregulate STAT3 to induce tumor invasion and migration (40). By contrast, as a tumor-suppressing lncRNA, PTCSC3 down-regulated STAT3 expression (41). To date, few studies identified LINC02555 and LINC01214 as the prognostic markers in cancer, their function and in-depth molecular interaction still remained unknown. The expression of LINC01214 appeared to have an effect on survival (42), still, more work is needed to explore the underlying mechanism. Some researchers also selected LINC01116 and LINC01671 into their prognostic model to predict survival (5). We further compared the prediction ability of this model with that of two published lncRNA signatures, and our signature had better prognostic performance. In addition, our lncRNA signature possessed prognostic value that was independent of TMB, age and pathologic stage.

Additionally, we found that there was a significantly higher ratio of patients with TP53 mutations in the high-risk group than in the low-risk group, implying that GILncSig could capture the TP53 mutation status. Previous research indicated that TP53 mutations are related to poorer survival in LUAD patients (43), and TP53-mutated tumors showed increased mutation burdens (44). In addition, we found that GILncSig could differentiate diverse clinical results in TP53 wild-type patients. In the low-risk group, TP53 wild-type patients had longer survival than TP53 mutation patients. However, no similar difference was observed in the high-risk group. The survival difference between low- and high-risk TP53 wild-type patients implied that GILncSig shows a superior prognostic correlation compared with TP53 mutation status alone. Similar results were found in EGFR wild-type and ALK wild-type patients (**Figures S1A, B**). Recent research has evaluated the prognostic role of TP53 and its correlation with EGFR mutation. They confirmed that TP53 mutation is a negative prognostic factor for NSCLC, and different affected exon would yield different prognostic value. Survival curve in EGFR mutation-negative patients indicated that TP53 mutation-negative patients had the best prognosis (45). Therefore, different TP53 mutations and multiple mutations may impact TP53 prognostic values. Considering coding mutations of TP53 occur relatively early in the development of lung cancer, usually before the tumor metastasizes (46), we speculated this is one of the reasons that TP53 show a superior prognostic value in low-risk patients. Furthermore, we found that some genes correlated with our candidate lncRNAs are also associated with cancer outcomes. CKMT (47) and MATN3 (48) were used to build risk models to predict the prognosis of cancer patients.

In conclusion, we used a mutation-derived approach to identify lncRNAs related to genomic instability, from which we built a 5-lncRNA signature (GILncSig), which was an independent prognostic marker, to stratify lung cancer patient risk subgroups. We further verified this result in the GEO database. However, since our study belongs to retrospective analysis, statistical power was hampered by possible selection bias such as driver mutations impact and other clinical confounders (e.g., smoking history, heterogeneous populations). We did not take them into consideration as this information was missing from public datasets. In this regard, our own cohort is warranted to validate their predictive role in lung cancer. Also, we only verify the differential expression of above-mentioned lncRNAs in lung cancer and normal tissues, in-depth molecular network of those lncRNAs and other factors still remained unknown, further works are needed to confirm the functions of those marker lncRNAs in lung cancer progress as well as their impact on patients’ survival. Hence, biological experiments based on lung cancer cell lines and animal model would be essential to explore the underlying mechanism and find new targets.

## Data Availability Statement

The datasets presented in this study can be found in online repositories. The names of the repository/repositories and accession number(s) can be found in the article/**Supplementary Material**.

## Ethics Statement

The studies involving human participants were reviewed and approved by Ethics Committee of Sun Yat-sen University Cancer Center. Written informed consent for participation was not required for this study in accordance with the national legislation and the institutional requirements.

## Author Contributions

Conception and design, LY, GG, and LZ. Administrative support, LZ, YL, YW, and WZ. Provision of study materials or patients, LY, GG, RZ, YY, and WX. Collection and assembly of data, GG, YW, RZ, GW, ZH, XZ, and DZ. Data analysis and interpretation, LY, GG, RZ, XY, DC, and WW. Manuscript writing, all authors. All authors contributed to the article and approved the submitted version.

## Conflict of Interest

The authors declare that the research was conducted in the absence of any commercial or financial relationships that could be construed as a potential conflict of interest.

## Publisher’s Note

All claims expressed in this article are solely those of the authors and do not necessarily represent those of their affiliated organizations, or those of the publisher, the editors and the reviewers. Any product that may be evaluated in this article, or claim that may be made by its manufacturer, is not guaranteed or endorsed by the publisher.
