# Supplementary material for: Mutation-Derived Long Noncoding RNA Signature Predicts Survival in Lung Adenocarcinoma
Source: Front Oncol. 2022 Mar 15;12:780631. doi: 10.3389/fonc.2022.780631 (PMC8965709; doi:10.3389/fonc.2022.780631)
Supplement: Supplementary file 1 [file DataSheet_1.docx]

**Supplementary materials**

**Figure S1**


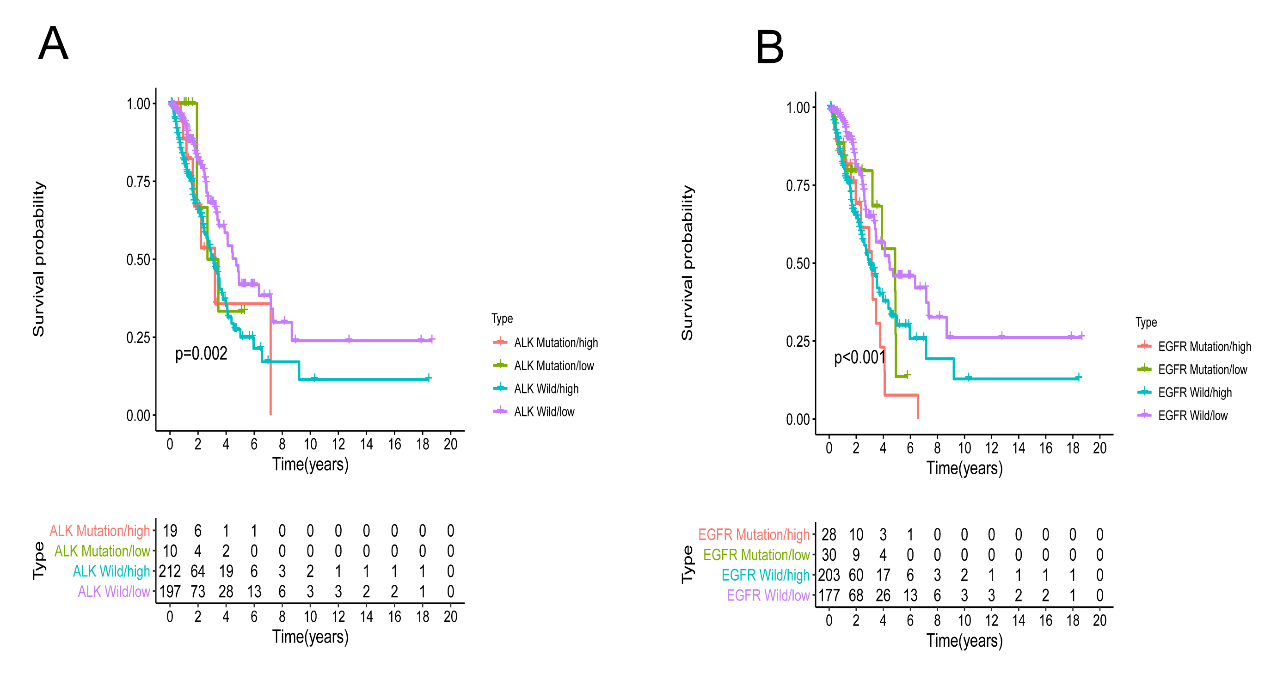


**Figure S1.** (A) Survival analysis of LUAD patients was categorized on the basis of the GILncSig and the ALK mutation status. (B) Survival analysis of LUAD patients was categorized on the basis of the GILncSig and the EGFR mutation status*.*

**Table S1. Differentially Expressed genes identified by using the “limma” R package**

| **lncRNA** | **conMean** | **Treat Mean** | **Log FC** | **p-Value** | **FDR** |
| --- | --- | --- | --- | --- | --- |
| AC000061.1 | 0.985859 | 0.161522 | -2.60965 | 0.000444 | 0.002213 |
| AL162511.1 | 4.882591 | 2.306255 | -1.08209 | 8.79E-11 | 5.49E-09 |
| PTCSC3 | 3.795601 | 1.893096 | -1.00358 | 3.84E-08 | 9.28E-07 |
| AC005856.1 | 0.88696 | 0.401984 | -1.14173 | 1.11E-07 | 2.13E-06 |
| AC010275.1 | 0.258685 | 0.5451 | 1.075323 | 0.000313 | 0.001653 |
| AC005479.2 | 1.73428 | 0.82688 | -1.06859 | 4.13E-08 | 9.86E-07 |
| AL445493.3 | 1.598361 | 0.371638 | -2.10462 | 2.68E-11 | 1.84E-09 |
| AL031058.1 | 0.99241 | 3.391138 | 1.772762 | 2.47E-16 | 5.25E-13 |
| AC012073.1 | 0.819953 | 1.782731 | 1.120476 | 2.06E-15 | 1.42E-12 |
| AP003119.2 | 0.616429 | 1.237623 | 1.005566 | 3.18E-05 | 0.000243 |
| UNC5B-AS1 | 0.664843 | 0.280771 | -1.24362 | 1.51E-08 | 4.02E-07 |
| AC010789.1 | 0.2297 | 1.597628 | 2.798109 | 8.44E-12 | 7.79E-10 |
| LINC01807 | 0.157786 | 0.661849 | 2.068533 | 8.23E-05 | 0.000543 |
| AL135999.3 | 6.721599 | 3.281955 | -1.03425 | 2.95E-06 | 3.25E-05 |
| LINC00922 | 2.532378 | 0.625413 | -2.01761 | 9.26E-08 | 1.87E-06 |
| LINC02446 | 1.13523 | 2.596371 | 1.193512 | 8.91E-07 | 1.21E-05 |
| AC090772.3 | 0.484364 | 1.000081 | 1.045953 | 0.001551 | 0.006055 |
| ATP13A4-AS1 | 2.1912 | 0.586311 | -1.90198 | 2.99E-05 | 0.00023 |
| LINC00665 | 2.44706 | 5.07963 | 1.053674 | 4.90E-12 | 5.66E-10 |
| LINC02159 | 1.323855 | 0.546079 | -1.27757 | 1.27E-10 | 7.47E-09 |
| FAM222A-AS1 | 0.248231 | 0.605615 | 1.286714 | 0.001295 | 0.005219 |
| AP003119.1 | 0.291358 | 0.703882 | 1.272542 | 0.000666 | 0.00306 |
| AC008268.1 | 8.097014 | 3.550414 | -1.1894 | 3.62E-07 | 5.66E-06 |
| LINC02555 | 1.475736 | 0.48825 | -1.59574 | 0.001668 | 0.006413 |
| AC099518.1 | 0.370852 | 0.962825 | 1.37643 | 6.89E-10 | 2.87E-08 |
| LINC01518 | 0.237826 | 0.945054 | 1.990491 | 1.98E-06 | 2.34E-05 |
| LINC01535 | 0.416798 | 0.876512 | 1.072426 | 1.07E-07 | 2.08E-06 |
| AC012213.4 | 0.204255 | 1.106916 | 2.438101 | 6.13E-06 | 6.08E-05 |
| RGMB-AS1 | 0.745392 | 0.366529 | -1.02407 | 1.55E-11 | 1.26E-09 |
| AL645924.1 | 1.37011 | 0.616175 | -1.15288 | 5.96E-06 | 6.00E-05 |
| AP003119.3 | 0.451739 | 1.225032 | 1.439258 | 3.55E-05 | 0.000265 |
| LINC02163 | 0.235553 | 0.893546 | 1.923491 | 1.27E-07 | 2.38E-06 |
| LINC02038 | 2.810243 | 0.939156 | -1.58126 | 1.99E-08 | 5.22E-07 |
| AC124312.3 | 0.90658 | 0.340179 | -1.41414 | 0.000187 | 0.001071 |
| LINC01194 | 0.102906 | 0.76877 | 2.901219 | 2.82E-06 | 3.14E-05 |
| LINC01765 | 0.959735 | 0.3193 | -1.58772 | 3.21E-09 | 1.13E-07 |
| AC020978.3 | 1.484818 | 0.636816 | -1.22134 | 0.000888 | 0.003905 |
| LINC00342 | 18.60177 | 6.435068 | -1.53141 | 1.36E-07 | 2.53E-06 |
| AC236972.3 | 0.807097 | 0.252577 | -1.67602 | 3.84E-13 | 7.41E-11 |
| SRGAP3-AS2 | 2.811144 | 0.968382 | -1.53751 | 2.94E-07 | 4.92E-06 |
| LINC01936 | 1.498404 | 0.680434 | -1.1389 | 1.98E-10 | 1.08E-08 |
| AL354993.2 | 0.266931 | 0.550421 | 1.044068 | 0.000759 | 0.003402 |
| AC120498.2 | 1.647454 | 0.569197 | -1.53324 | 0.000527 | 0.002546 |
| BCAR4 | 0.330699 | 1.533922 | 2.213637 | 7.16E-08 | 1.51E-06 |
| TRPM2-AS | 0.350796 | 0.892057 | 1.346503 | 0.001965 | 0.007299 |
| AC019171.1 | 0.263612 | 1.872581 | 2.828538 | 9.76E-06 | 8.86E-05 |
| AC010998.3 | 0.97452 | 0.236335 | -2.04386 | 2.45E-07 | 4.23E-06 |
| AC011379.2 | 0.607508 | 0.285616 | -1.08882 | 6.03E-06 | 6.04E-05 |
| LINC01088 | 1.196029 | 0.155188 | -2.94616 | 0.00013 | 0.000798 |
| AC091588.1 | 0.690241 | 0.259052 | -1.41386 | 1.21E-06 | 1.55E-05 |
| LINC01644 | 1.489985 | 0.588872 | -1.33927 | 1.74E-11 | 1.37E-09 |
| AP005131.3 | 0.665185 | 0.300867 | -1.14463 | 0.000435 | 0.002178 |
| LINC02195 | 0.838675 | 1.758265 | 1.067969 | 1.94E-06 | 2.31E-05 |
| ERVE-1 | 0.691455 | 0.322949 | -1.09833 | 4.04E-09 | 1.38E-07 |
| MIR3142HG | 1.320971 | 0.655526 | -1.01087 | 7.75E-07 | 1.07E-05 |
| AC074135.1 | 0.656029 | 1.419282 | 1.113329 | 0.002812 | 0.009804 |
| C8orf34-AS1 | 4.886385 | 1.734515 | -1.49424 | 5.85E-14 | 1.99E-11 |
| LINC01395 | 0.266715 | 0.618165 | 1.212691 | 5.52E-08 | 1.23E-06 |
| LINC01671 | 3.208679 | 1.372026 | -1.22567 | 1.54E-09 | 6.05E-08 |
| AL133370.1 | 2.203023 | 1.068968 | -1.04327 | 0.001089 | 0.004589 |
| LINC00346 | 0.724499 | 1.728386 | 1.25437 | 1.43E-08 | 3.88E-07 |
| MIR193BHG | 0.527214 | 1.185616 | 1.169177 | 1.49E-07 | 2.72E-06 |
| AC013275.1 | 1.413821 | 0.637529 | -1.14904 | 2.32E-08 | 5.93E-07 |
| POU6F2-AS2 | 0.396787 | 0.882522 | 1.153267 | 1.51E-05 | 0.00013 |
| ELFN1-AS1 | 0.23983 | 1.668893 | 2.798805 | 4.57E-08 | 1.07E-06 |
| LINC01980 | 0.213523 | 1.597256 | 2.903133 | 3.68E-07 | 5.68E-06 |
| AC104031.1 | 3.172157 | 0.65271 | -2.28095 | 2.14E-11 | 1.57E-09 |
| DIO3OS | 0.758669 | 0.181293 | -2.06515 | 2.67E-15 | 1.42E-12 |
| AL355601.1 | 0.746552 | 0.281204 | -1.40862 | 1.19E-05 | 0.000106 |
| LINC01224 | 0.285594 | 1.145747 | 2.004252 | 1.78E-05 | 0.00015 |
| EGOT | 0.952993 | 0.415246 | -1.1985 | 4.61E-09 | 1.48E-07 |
| FAM83A-AS1 | 1.398995 | 4.068282 | 1.540029 | 1.16E-06 | 1.51E-05 |
| GATA6-AS1 | 0.919123 | 0.187765 | -2.29133 | 5.38E-11 | 3.57E-09 |
| LINC01942 | 0.831017 | 0.256451 | -1.69619 | 3.07E-14 | 1.30E-11 |
| LINC01133 | 3.940608 | 1.429526 | -1.46288 | 7.34E-05 | 0.000489 |
| ELN-AS1 | 5.041841 | 2.341575 | -1.10647 | 4.92E-13 | 8.70E-11 |
| AL355388.1 | 1.201066 | 0.458096 | -1.39059 | 8.42E-11 | 5.42E-09 |
| AP005233.2 | 0.984964 | 2.759607 | 1.48632 | 0.000109 | 0.000686 |
| UBXN10-AS1 | 2.936548 | 1.299366 | -1.17631 | 3.76E-10 | 1.81E-08 |
| AC006213.3 | 0.314573 | 0.646145 | 1.038464 | 3.27E-07 | 5.30E-06 |
| AC005479.1 | 1.349387 | 0.65963 | -1.03258 | 4.86E-07 | 7.23E-06 |
| LINC01550 | 0.56442 | 0.248224 | -1.18513 | 9.04E-13 | 1.37E-10 |
| AC006206.2 | 0.39007 | 0.8852 | 1.18227 | 2.62E-05 | 0.000205 |
| AC116345.1 | 0.636235 | 0.221467 | -1.52247 | 2.43E-05 | 0.000194 |
| RHOXF1-AS1 | 2.210763 | 0.791455 | -1.48196 | 1.22E-11 | 1.04E-09 |
| LINC00648 | 0.230893 | 0.775807 | 1.748474 | 0.002664 | 0.009396 |
| LINC01504 | 1.132549 | 0.47614 | -1.25012 | 2.47E-10 | 1.25E-08 |
| AC025154.2 | 2.789983 | 0.909231 | -1.61754 | 2.29E-11 | 1.62E-09 |
| COLCA1 | 4.341958 | 1.929598 | -1.17005 | 6.53E-09 | 2.00E-07 |
| FENDRR | 1.01621 | 0.365109 | -1.4768 | 1.01E-07 | 1.98E-06 |
| AL357093.2 | 2.433788 | 0.928128 | -1.39081 | 2.82E-06 | 3.14E-05 |
| LINC01612 | 1.8167 | 0.352101 | -2.36726 | 6.11E-13 | 9.98E-11 |
| LINC02588 | 0.322862 | 0.744731 | 1.2058 | 0.000515 | 0.002513 |
| AL021807.1 | 0.663946 | 1.460604 | 1.137428 | 1.63E-05 | 0.000139 |
| KCNMB2-AS1 | 0.898085 | 1.925014 | 1.099945 | 0.000586 | 0.00274 |
| LINC02587 | 0.876662 | 0.084166 | -3.38071 | 5.92E-09 | 1.85E-07 |
| LINC00337 | 0.218148 | 0.772125 | 1.823528 | 7.49E-14 | 1.99E-11 |
| CASC9 | 1.528157 | 3.399145 | 1.153379 | 6.18E-05 | 0.000422 |
| SOCS2-AS1 | 0.854118 | 0.421332 | -1.01948 | 4.12E-06 | 4.42E-05 |
| AC110741.1 | 1.701644 | 0.224907 | -2.91953 | 8.84E-06 | 8.20E-05 |
| AC003092.1 | 0.09306 | 1.702648 | 4.193482 | 0.000578 | 0.002719 |
| AC016877.3 | 0.300436 | 0.959618 | 1.675403 | 4.16E-12 | 5.20E-10 |
| LHFPL3-AS2 | 5.40019 | 0.850748 | -2.66621 | 1.69E-15 | 1.42E-12 |
| AP001107.9 | 2.313629 | 1.017173 | -1.18559 | 1.48E-10 | 8.50E-09 |
| AL079303.1 | 0.341464 | 0.732298 | 1.100695 | 0.000128 | 0.000784 |
| SFTA1P | 38.26587 | 10.77093 | -1.82892 | 1.19E-11 | 1.04E-09 |
| AC011944.1 | 1.154975 | 0.271514 | -2.08876 | 5.33E-12 | 5.66E-10 |
| AC089983.1 | 0.352584 | 2.129032 | 2.59416 | 9.35E-07 | 1.26E-05 |
| AL136162.1 | 0.315389 | 0.633288 | 1.00573 | 3.22E-06 | 3.51E-05 |
| BX640514.2 | 1.296339 | 0.63536 | -1.0288 | 1.15E-10 | 6.98E-09 |
| XIST | 6.369446 | 2.977267 | -1.09718 | 0.002146 | 0.007832 |
| AC131009.1 | 0.346406 | 0.722335 | 1.060202 | 2.54E-05 | 0.0002 |
| AL139023.1 | 0.146949 | 0.814837 | 2.471192 | 1.45E-06 | 1.79E-05 |
| AC026785.3 | 0.560676 | 2.195731 | 1.969463 | 0.001638 | 0.006358 |
| LINC00524 | 0.761474 | 0.219968 | -1.7915 | 2.90E-09 | 1.04E-07 |
| AL109615.3 | 1.038941 | 4.651733 | 2.162654 | 1.21E-13 | 2.85E-11 |
| AC026369.3 | 1.358248 | 0.635655 | -1.09543 | 0.00121 | 0.004935 |
| LINC02122 | 0.83593 | 0.246451 | -1.76208 | 9.15E-08 | 1.87E-06 |
| AC061975.6 | 0.129515 | 0.767985 | 2.56796 | 4.26E-06 | 4.54E-05 |
| PSORS1C3 | 2.744187 | 1.286826 | -1.09256 | 0.000108 | 0.000684 |
| PARAL1 | 1.113423 | 0.517261 | -1.10604 | 1.25E-06 | 1.60E-05 |
| LINC01214 | 0.359614 | 1.389918 | 1.950479 | 4.29E-06 | 4.56E-05 |
| LINC00942 | 1.125366 | 6.246302 | 2.472608 | 6.22E-06 | 6.08E-05 |

**Table S2. Univariate Cox regression analyses of genome instability-associated lncRNAs related to overall survival in LUAD**

| **LncRNA** | **HR** | **HR.95L** | **HR.95H** | **p-value** |
| --- | --- | --- | --- | --- |
| PTCSC3 | 0.887189 | 0.798635 | 0.985561 | 0.025678 |
| LINC02555 | 0.708696 | 0.515377 | 0.97453 | 0.034114 |
| LINC01671 | 1.033681 | 1.002912 | 1.065394 | 0.031668 |
| LINC00346 | 1.137948 | 1.021997 | 1.267055 | 0.018434 |
| MIR193BHG | 1.265632 | 1.013933 | 1.579813 | 0.037318 |
| LINC01116 | 1.145254 | 1.051395 | 1.247492 | 0.001879 |
| AC003092.1 | 1.015629 | 1.000697 | 1.030784 | 0.040149 |
| LINC01214 | 1.088377 | 1.027751 | 1.152579 | 0.003779 |

**Table S3. Sequences of primers used for qRT-PCR**

| **Gene** | **Primer** | **Sequence** |
| --- | --- | --- |
| PTCSC3 | Forward | CATGCCTCCTCATAATCATATCTTC |
|  | Reverse | TGCTACTGTGAGCATAACCTACTTAC |
| LINC02555 | Forward | AGGGCTCTCTGTGATCTGGC |
|  | Reverse | GAGGTTGTCGGGGAGCTTTC |
| LINC01671 | Forward | TTCTGGTGAACTGAGGAGGGA |
|  | Reverse | GCTTGCTGAGACTGGAGTATGG |
| LINC01116 | Forward | CTAAGAATGGGTCTCACTCTGC |
|  | Reverse | CTCGGAAGGCTGAGGTGGGAG |
| LINC01214 | Forward | CTGGTAGCAATGGGGACACG |
|  | Reverse | AGCAGCAACACTCTTCACCG |
| β-Actin | Forward | CACCATTGGCAATGAGCGGTTC |
|  | Reverse | AGGTCTTTGCGGATGTCCACGT |
